# Volume XXIII

**Published:** 1883-08

**Authors:** 


### Editorial.

### Volume XXIII.

The present number introduces to our numerous subscribers and readers the twenty-third volume of the Journal, the fifth under the present editorial management. What we have had occasion to say heretofore of the success of this enterprise in medical journalism may well be repeated here, that this Journal is a reflex of the activity and zeal of the profession of this city rather than the product of our personal efforts, or a measure of our editorial ability. Judged by this standard, the increased value of the last volume affords an encouraging indication that Buffalo in its professional interests and activity is keeping pace with its great commercial progress which has received an impetus within the past few years unparalleled in its history.

Taking advantage of this increased activity in commercial as well as professional circles, we enter upon the work of another volume, with an enthusiasm born of past success and inspired by surrounding circumstances, while we have reason to be confident that under such improved auspices the JOURNAL will be able to meet with greater favor among its patrons, and continue to merit the favorable opinion and judgment of the profession. The constant increase in our subscription list, now larger than at any time since the present editors assumed charge of it, is a flattering indication of the kindly reception given to the JOURNAL wherever it is known and read.

For the future it is our purpose to give new value to its pages by publishing the best clinical lectures we are able to secure, with original communications from the ablest writers in the profession, and we invite our readers to furnish reports of rare and interesting cases occurring in daily practice. These features give a strictly scientific value to our pages, with a practical importance which will suit the more laborious scientific student and also the general practitioner.

The reputation always maintained by the JOURNAL places a responsibility upon the editors which it will be their effort to meet, and we rely upon the co-operation of the profession for such assistance as both opportunity and inclination may afford.

